# Improving birth preparation with the hypnosis online course “The Peaceful Birth”: a randomized controlled study

**DOI:** 10.3389/fpsyg.2025.1508790

**Published:** 2025-01-20

**Authors:** Luisa Motz, Rosa Marie Brückner, Barbara Schmidt

**Affiliations:** ^1^Institute for Psychology, Friedrich-Schiller-University Jena, Jena, Germany; ^2^Department of Geriatrics, Halle University Hospital, Halle, Germany; ^3^Institute for Psychosocial Medicine, Psychotherapy and Psychooncology, Jena University Hospital, Jena, Germany

**Keywords:** hypnosis, childbirth, birth preparation, birth experience, birth expectation, fear of birth, birth pain

## Abstract

**Objective:**

Stress, anxiety, and birth-related expectations affect the way women experience birth. In this study, we evaluate the German online hypnosis program “The Peaceful Birth” (“Die Friedliche Geburt”) to find out if it reduces stress, anxiety, and pain during pregnancy. We randomized pregnant women into a hypnosis and a control group and surveyed them via online questionnaires during their pregnancy and after their birth using standardized questionnaires.

**Methods:**

We included 221 pregnant women and assigned 110 women to a hypnosis and 111 women to a control group. At four measurement points before and after birth, we surveyed all study participants. We used the Perceived Stress Scale to measure stress experiences and the Wijma Delivery Expectancy/Experience Questionnaire to assess childbirth expectations and experiences.

**Results:**

We show that stress was significantly reduced in the hypnosis group before and after birth compared to the control group (*p* = 0.019) and childbirth expectations were significantly more positive compared to the control group after starting the hypnosis online course (*p* = 0.002). Subjective health ratings in the hypnosis group improved significantly compared to the control group before childbirth (*p* = 0.002). Furthermore, women in the hypnosis group described childbirth experience as significantly more positive concerning fear (*p* = 0.007), loneliness (*p* = 0.025), and self-efficacy (*p* = 0.047) compared to the control group. We show that the improvement of childbirth experience is causally related to more positive childbirth expectations. However, we found no significant group differences in the experience of pain (*p* = 0.08).

**Conclusion:**

Pregnant women preparing for birth with the hypnosis online course “The Peaceful Birth” showed significantly improved birth preparation showing lower stress, more positive birth expectation and experience compared to a control group. The hypnosis online course is therefore an efficient low-threshold method to enhance healthcare for expectant mothers.

## Introduction

Childbirth is a unique and intense experience for expectant mothers. It significantly affects maternal physical and mental health, coping with motherhood, and mother–child bonding (Beech and Phipps, 2008). About a quarter of mothers describe their experience of childbirth as negative or even traumatic (Larsson et al., 2011; Hosseini Tabaghdehi et al., 2019). Various factors can influence the experience of childbirth. Initially, the anticipation of childbirth with prevailing expectations plays a crucial role (Conrad and Trachtenberg, 2021). Research suggests that a more positive anticipation of childbirth leads to greater satisfaction with the childbirth experience. Negative expectations can lead to fear of birth, which is associated with a more negative childbirth experience (Ayers and Pickering, 2005; Conrad and Trachtenberg, 2021). Another important factor is the mindset of birth that predicts birth outcomes (Hoffmann et al., 2023). Fear of birth exists on a continuum ranging from mild concerns to pronounced pathological tokophobia (Richens et al., 2018). Several studies highlight the widespread prevalence of fear of birth in moderate to pathological severity among pregnant women (Demšar et al., 2018; O’Connell et al., 2017). A pronounced fear of birth with prevailing negative cognitions is problematic in accordance with the transactional stress model as it causes women to perceive the upcoming birth as a threatening situation that is hard to cope with (Lazarus and Folkman, 1984). If the expectant mother perceives limited resources and abilities to overcome the situation, this results in an increased experience of stress. Antonovsky’s salutogenesis model describes a similar relationship, according to which health and well-being result from a strong sense of coherence. Coherence is associated with the perception of a situation as manageable, meaningful and comprehensive (Antonvsky et al., 1997). If a pregnant woman perceives to have all necessary resources for giving birth, this results in a strong sense of coherence, which helps her to manage the situation (Prinds et al., 2022).

Another important factor influencing the birth experience is the perception of pain during labor. Although there is great variability in the perception of birth pain (Lowe, 2002) many women describe the pain of vaginal birth as extremely intense (Dahan, 2023). Physiologically, labor pain is caused by the severe dilation of the cervix, tension on the uterine ligaments, stretching of the pelvic floor, perineum and vulva and tissue damage due to ischemia (Lowe, 2002). However, psychological factors, such as fear of childbirth, also influence the intensity of labor pain. The Fear-Tension-Pain cycle, postulated by Dick-Read (1972) describes this relationship as follows: Fear leads to activation of the sympathetic nervous system, which in turn leads to increased muscle tension, vasoconstriction and reduced oxygen supply to the uterus. As a result, labor pain is increased.

A method that has been well-studied for its efficacy in reducing anxiety and pain is hypnosis. According to a meta-analysis by Thompson et al. (2019), hypnosis shows moderate to high efficacy in providing pain relief. Hypnoanalgesia refers to the use of hypnosis to reduce or manage pain. It is a non-pharmacological technique that can be employed to alleviate both acute and chronic pain by altering the individual’s perception of pain or their emotional and physiological response to it. Hypnoanalgesia effectively reduces pain intensity, pain tolerance and pain threshold. In terms of anxiety reduction, hypnosis is effective in reducing preoperative anxiety (Saadat et al., 2006), anxiety during dental treatment (Glaesmer et al., 2015), and claustrophobia (Napp et al., 2021). Hypnosis is also effective in reducing stress (Schmidt et al., 2024) and anxiety during medical procedures such as non-invasive ventilation (Schmidt et al., 2021; Schmidt, 2024). Even physical parameters like handgrip strength can be increased via hypnosis (Nieft et al., 2024).

In recent years, hypnosis has gained popularity as a method for childbirth preparation (Fernandez-Gamero et al., 2024). An increasing number of guides and applications promote hypnobirthing as a method that improves the birth experience. However, some of the methods promoted as hypnobirthing include esoterically connoted, insufficiently studied practices. Yet, there are already many scientific publications that prove the efficacy of evidence-based hypnosis for birth preparation (Catsaros and Wendland, 2023). A systematic review by Catsaros and Wendland (2023) revealed that hypnosis-based interventions effectively reduce anxiety during childbirth and improve feelings of control. Consequently, these interventions contributed to an improved emotional experience and positively influenced women’s attitudes toward childbirth. This positive shift resulted in reduced fear, heightened satisfaction, fewer birth interventions, improved postnatal well-being, and an overall enhanced birth experience. Moreover, a study by Abbasi et al. (2009) demonstrated that women participating in hypnosis-based interventions are more likely to reinterpret sensations of pain during labor as sensations of pressure. This reinterpretation promotes more positive thoughts and the birth process is experienced as more satisfying.

One commercially available birth preparation program that utilizes audio hypnosis is the online course “The Peaceful Birth” (in German: “Die Friedliche Geburt”), developed by Kristin Graf. This online course aims to enhance attitudes toward childbirth and ultimately improve the childbirth experience by using positive suggestions. The course focuses on strengthening confidence in one’s coping abilities and aims to reduce fear, stress and birth pain. Please note that it was not suggested that there will be no pain during labor. Instead, the program helps pregnant females to better cope with pain.

The objective of this study is to assess the efficacy of the hypnosis-based intervention “The Peaceful Birth.” To do this, we measured birth expectation, birth experience and the associated factors of stress, pain and well-being. We expected that the intervention would reduce stress and fear, leading to an overall more positive birth expectation and an improved sense of health and well-being. We also expected that the birth experience itself would be improved and that birth pain would be reduced. We furthermore hypothesized that the improvement in birth expectation and birth experience would be causally related. In addition to these factors, we also looked at whether taking part in the intervention had an effect on birth costs and newborn health parameters.

### Role of the funding source

The authors had access to relevant aggregated study data and other information (such as study protocol, analytic plan and report, validated data table, and clinical study report) required to understand and report research findings. The authors take responsibility for the presentation and publication of the research findings, have been fully involved at all stages of publication and presentation development, and are willing to take public responsibility for all aspects of the work. All individuals included as authors and contributors who made substantial intellectual contributions to the research, data analysis, and publication or presentation development are listed appropriately. The role of the sponsor in the design, execution, analysis, reporting, and funding is fully disclosed. The authors’ personal interests, financial or non-financial, relating to this research and its publication have been disclosed.

## Methods

### Participants

We conducted an *a priori* power analysis using G*Power (Faul et al., 2007). Our primary outcome was perceived stress, as we expected reduced stress ratings in the hypnosis group compared to the control group. To estimate the expected effect size in a between-group *t*-test, we referred to a meta-analysis investigating the efficacy of hypnosis in surgical procedures (Holler et al., 2021). This meta-analysis estimated a medium-sized effect of hypnosis on mental distress. We calculated that we need a minimum of 102 participants to detect an effect size of *d* = 0.5 with *α* = 0.05 and *β* = 0.20. To account for a potential dropout during the four measurement time points, we aimed to recruit 200 participants. We included all participants who were at least 18 years old and pregnant in the second trimester, between week 13 and 28 of pregnancy. Furthermore, we only included women who were not planning to attend or were already enrolled in a prenatal hypnosis course, because we wanted to measure the effect of our specific online hypnosis intervention.

**Figure 1:**
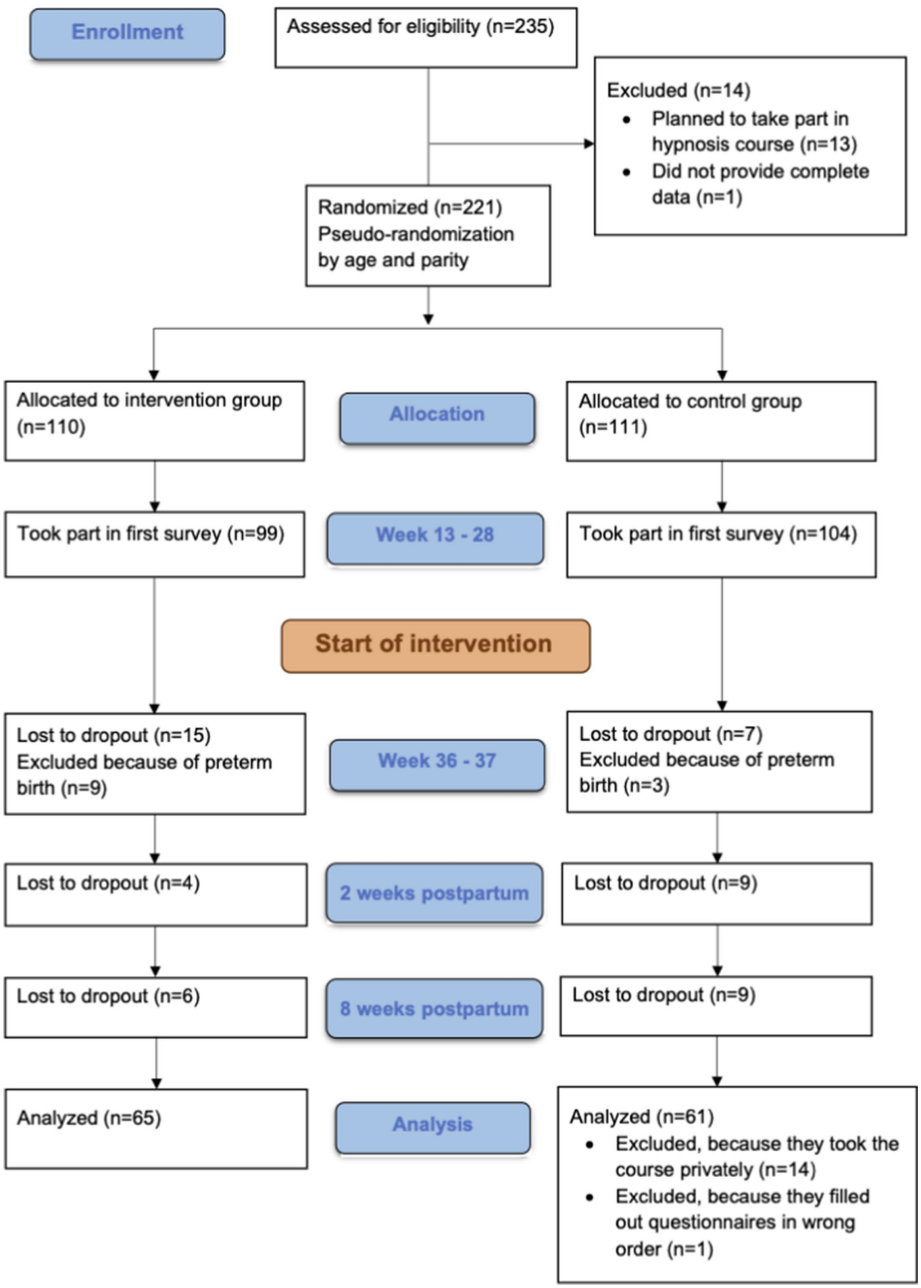
Study flow chart.

We recruited participants via a newsletter advertisement on “Babelli.de,” a website that provides information on pregnancy, childbirth and infants. We chose Babelli.de as recruiting platform to make sure we reach pregnant women who are generally interested in interventions to improve their pregnancy but not especially hypnosis. In the advertisement, we told the pregnant females that we will support them during pregnancy by providing questionnaires and called the study “the good birth.” The recruited sample consisted of 221 pregnant women. They were randomized into a hypnosis and control group. We kept age and parity equal in both groups, as these factors play a role in birth expectation and birth experience (Aasheim et al., 2013; Booth and Meltzoff, 1984). Older women have an increased risk of adverse birth outcomes and older women experiencing their first pregnancy even more (Lisonkova et al., 2010). Due to dropout during the four measurement time points, the sample was reduced by 68 women. In addition, 12 women were excluded who had already given birth before the second measurement, 14 women of the control group were excluded because they had attended the hypnosis online course privately and 1 woman was excluded because she completed the third questionnaire before the second. As a result, the final analysis included 65 women in the intervention group and 61 women in the control group. See Figure 1 for a flow chart of the study.

The women in the final sample were on average *M* = 32.98 (*SD* = 3.96) years old and had previously given birth to *M* = 0.29 (*SD* = 0.61) children. Participants in both groups had on average barely previous experience with hypnosis.

We included both women who had their babies in hospitals and women who had their babies in other places, such as birth centers or at home. In our sample, 7 women gave birth at home (2 in the hypnosis group, 5 in the control group) and 4 women gave birth in birth centers (2 hypnosis group, 2 control group). All other women gave birth in hospitals.

All women gave informed consent for participating in the study. As an expense allowance for participating in the study, all women received access to a postnatal online course. This postnatal course was provided by www.glücksmama.de and is called “Mehr als Rückbildung.”

### Procedure

The study had a longitudinal design with four measurement time points. The first measurement (between week 13 and 28 of pregnancy) served as a baseline measurement, the second measurement (between week 36 and 37 of pregnancy) focused on the anticipation of childbirth, the third measurement (2 weeks after the expected date of delivery) explored the birth experience and the fourth measurement (8 weeks after the expected date of delivery) examined the entry into life with a newborn.

In addition, there was a pre-survey to pseudo-randomize the women into intervention and control group, and a post-survey to measure constructs that were not initially considered at the time of study conception. The post-survey was sent to the participants after the fourth measurement point as soon as possible. At each measurement point, women received a questionnaire link via e-mail. All questionnaires were developed using Soscisurvey software. If participants had not completed the questionnaire within 3 days, we sent a one-time reminder email. Within our questionnaires, we used the standardized instruments Wijma Delivery Expectancy/Experience Questionnaire (Wijma et al., 1998) and Perceived Stress Scale (Cohen et al., 1983). All questionnaires can be accessed via the following link: https://cloud.uni-jena.de/s/2GtmNWfi9dpmF2D.

**Figure 2:**
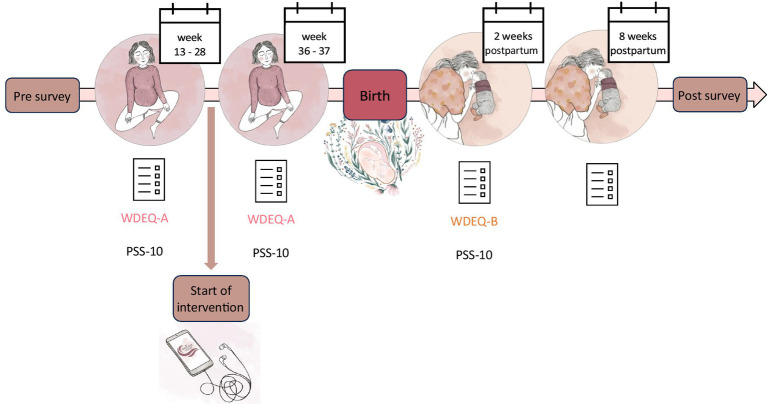
Experimental design.

After the first measurement, women in the hypnosis group received access to the hypnosis online course “The Peaceful Birth” for independent use. We assessed the intensity of program usage at the subsequent three measurement points as a control measure. Women in the control group received no intervention. An overview of the study procedure is displayed in Figure 2.

The study was approved by the Ethics Committee of the Jena University Hospital on September 6, 2022 under the reference number 2022-2709-BO. As part of our commitment to transparency in research, we pre-registered the study with the German Clinical Trials Register (DRKS) on April 4, 2023 under the registration ID DRKS00031618. The pre-registration can be viewed at: https://drks.de/search/en/trial/DRKS00031618.

### Materials

#### Hypnosis online course

The hypnosis online course “The Peaceful Birth” was provided free of charge to participants in the hypnosis group for individual use. The course is accessible through a website and a mobile app. Its core content comprises 65 sessions of audio hypnosis, categorized into hypnosis for practice, during childbirth, special circumstances, postnatal, and universal hypnosis. Complementing this, four video modules provide knowledge transfer on childbirth and hypnosis, covering aspects such as the physical and psychological processes of childbirth, the mechanics of hypnosis, and the autohypnosis method, that is taught in the course. Supporting materials, including an exercise plan to guide the selection of appropriate audio hypnosis at different stages, accompany the course contents. Additionally, regular online question and answer sessions, along with practice groups, are available for course participants.

#### Questionnaires

We used group-specific questionnaires for each of the four measurement time points. The first measurement covered sociodemographic variables, prior birth experiences, expectations of hypnosis and a standardized assessment of childbirth expectations and stress. The second measurement included questions on the progress of the current pregnancy, health, expectations of hypnosis efficacy and again a standardized measurement of birth expectations and stress. Additionally, the hypnosis group received queries on intervention use and perceived benefits. The third measurement focused on the childbirth experience itself, including pain and health related questions. Again, standardized questionnaires were used, this time assessing stress and birth experience. We asked the hypnosis group repeatedly about intervention use and benefits. The fourth measurement evaluated women’s health perception, well-being, and transition into postnatal life, with added questions on childbirth costs. Hypnosis group members were questioned once more on course use and perceived benefits.

In a follow-up survey, we measured general self-efficacy and childbirth-related mindset using the German versions of the ASKU (General Self-Efficacy Scale - Short Form) (Beierlein et al., 2013) and the Mindset and Birth Questionnaire (Hoffmann and Banse, 2021). The ASKU scale consists of three items, measuring subjective competence expectations on a 5-point Likert scale (Beierlein et al., 2013). The Mindset and Birth Questionnaire, assessing the natural or medical orientation of childbirth- related mindset, includes 18 items on a 6-point Likert scale (Hoffmann and Banse, 2021).

#### Standardized measurement of birth expectation and birth experience

To measure birth expectation and birth experience, we used the German version (Hansche et al., 2009a,b) of the Wijma Delivery Expectancy/Experience Questionnaire (WDEQ) in version A (Childbirth Expectancy) and B (Childbirth Experience) (Wijma et al., 1998). This questionnaire is commonly used to assess childbirth-related fears. WDEQ-A is provided before birth to measure birth expectancy, WDEQ-B is provided after birth to measure birth experience. For our study, we used version A at the first and second measurement point and version B at the third measurement point. Both versions have 33 equivalent items to be answered on a 5-point Likert scale. Consequently, a total score ranging from 0 to 165 can be obtained, with higher scores indicating a more negative childbirth expectation or experience.

Originally, the questionnaire was designed as unidimensional, yet the German version of the WDEQ-B underwent factor analysis revealing the presence of six factors: “Lack of self- efficacy,” “Loneliness,” “Negative appraisal,” “Fear,” “Negative experience,” and “Not as it should be” with internal consistencies ranging from *α* = 0.71 to *α* = 0.82 (König, 2019).

#### Standardized measurement of stress

For assessing stress, we used the German version (Schneider et al., 2020) of the Perceived Stress Scale (PSS-10) (Cohen et al., 1983) at the first three measurement points. This scale measures the extent to which individuals perceived situations in the past month as overwhelming, uncontrollable, or unpredictable compared to their individual coping abilities. The 10 items are responded to on a 5-point Likert scale. The scale demonstrates good validity and reliability, with an internal consistency ranging from *α* = 0.88 to *α* = 0.89.

### Statistical analyses

For all statistical analyses we used R version 4.3.1 (R Core Team, 2023). We provide open access to the analysis scripts via Zenodo.^1^

We analyzed all variables that we measured multiple times via mixed within-between ANOVAs. These variables are perceived stress, birth expectation and health ratings. We expected that stress ratings will be lower in the hypnosis group, that birth expectation will be more positive in the hypnosis group and that health ratings will be better in the hypnosis group compared to the control group. In addition to the ANOVAs, we also calculated one-tailed between-group *t*-tests for these variables for each measurement point.

For variables that we measured on only one measurement time, we calculated one-tailed between-group *t*-tests. We expected that birth experience will be better in the hypnosis group and that pain ratings will be lower in the hypnosis group compared to the control group. Where it was appropriate, we included covariates in the calculation. Furthermore, we conducted mediation analyses for birth expectation and birth experience subscales.

We measured exploratory variables where we did not have directed hypotheses. We investigated birth costs as reported by our participants, neonatal health scores from participants’ maternity passport and data from the ASKU and the Mindset and Birth questionnaire. We conducted two-tailed between-group *t*-tests for these variables as we did not expect group effects here.

Additionally, we calculated correlations within the hypnosis group between intensity of course use and perceived benefits as we expected that more intense course usage predicts better outcomes.

## Results

### Sample characteristics

In Table 1, we sum up the characteristics of the two groups. Please note that we randomized our participants keeping age and parity similar in both groups, so we did not have significant differences between groups.

**Table 1:** Sample characteristics.

	Means and standard deviations	
	Hypnosis group (*n* = 65)	Control group (*n* = 61)
Age	32.68 (sd = 3.36)	33.28 (sd = 4.49)
Parity	0.20 (sd = 0.47)	0.39 (sd = 0.71)

### Stress

**Figure 3:**
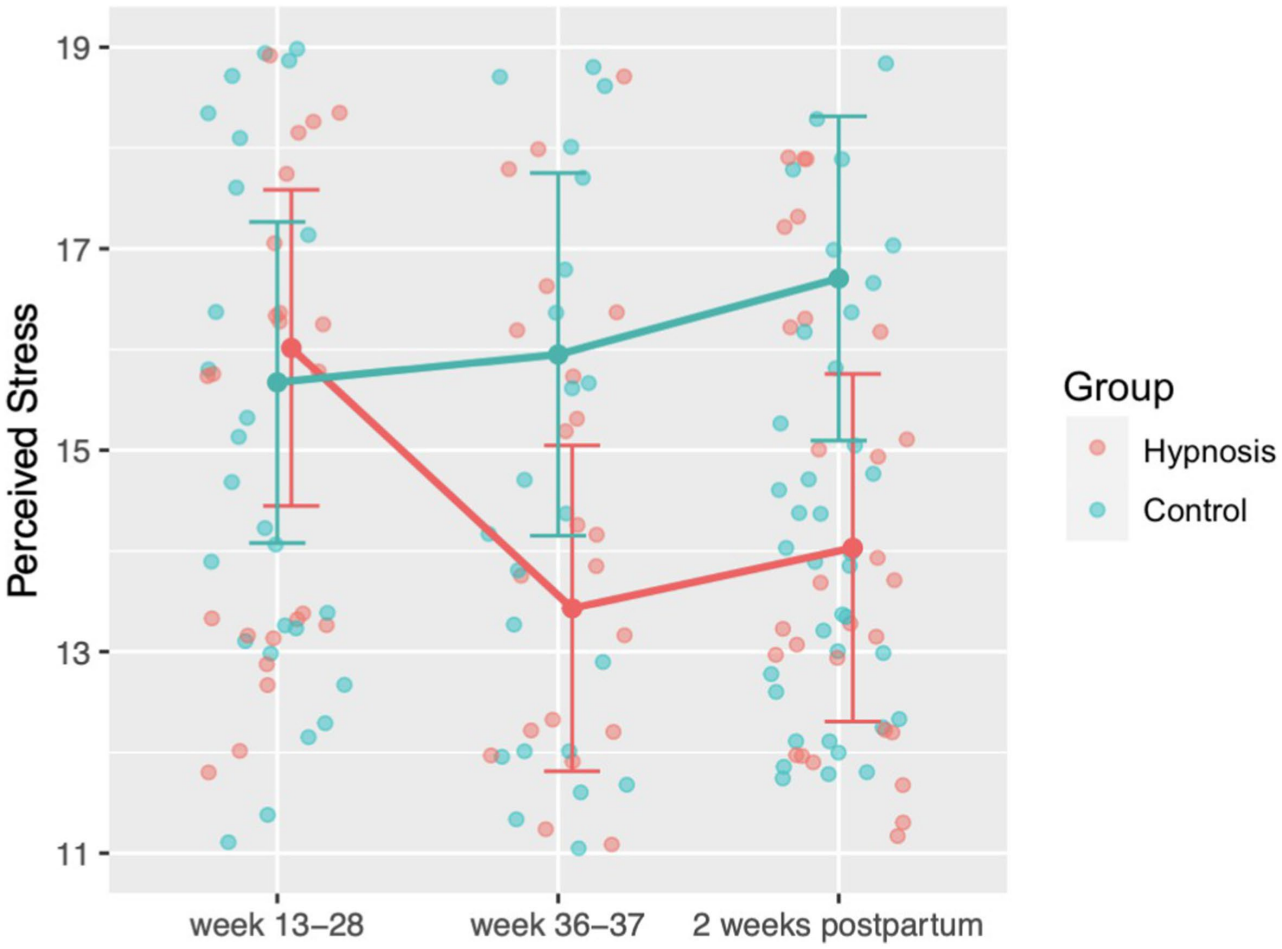
Perceived stress ratings before the start of the hypnosis online course (week 13–28) until 2 weeks after birth, measured with the PSS-10 questionnaire. After the start of the hypnosis intervention, women in the hypnosis group felt significantly less stressed compared to the control group.

To analyze participants’ stress experience, we used the data of the Perceived Stress Scale and calculated a mixed ANOVA with the factors group (hypnosis, control) and time point (week 13–28 of pregnancy, week 36–37 of pregnancy, 2 weeks after birth). We used a Greenhouse–Geisser correction for the analysis as the sphericity assumption was violated. We found a significant interaction effect between group and time point *F*(2, 246) = 5.30, *p* = 0.006 (see Figure 3). Post-hoc comparisons revealed that stress experience decreased significantly for women in the hypnosis group from the first to the second time point *t*(123) = 4.04, *p* = 0.001. Analyzing the group differences after the start of the hypnosis intervention at the second and third time point, we found a significant main effect of group *F*(1,123) = 5.62, *p* = 0.019, showing that women in the hypnosis group experienced significantly less stress than women in the control group at both time points.

### Birth expectation and birth experience

**Figure 4:**
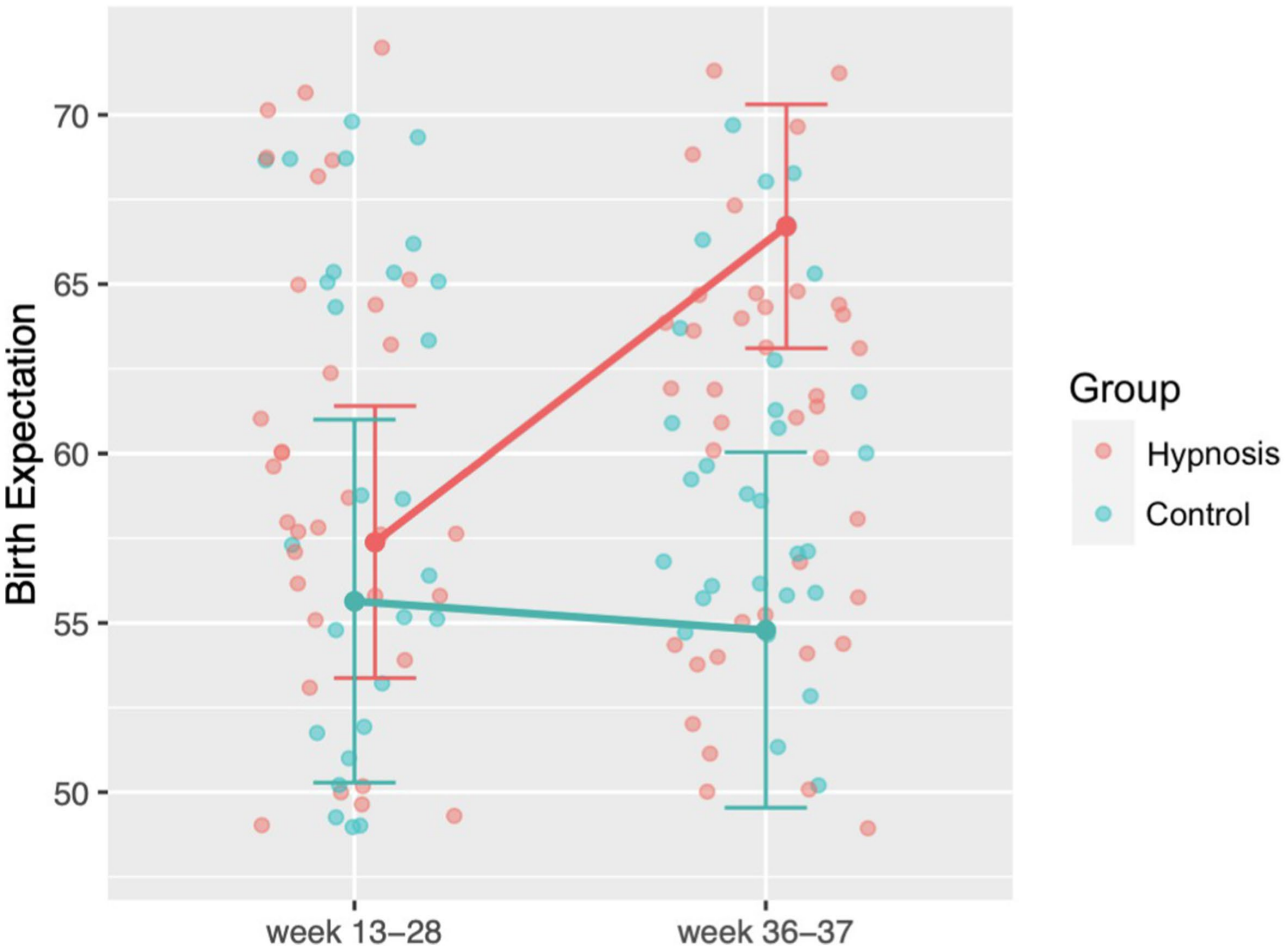
Changes in birth expectation during pregnancy measured with the WDEQ-A questionnaire. After the start of the hypnosis intervention, women in the hypnosis group showed significantly more positive birth expectations compared to the control group. For facilitating the readability, we transformed WDEQ values so that higher values indicate a more positive birth expectation.

We analyzed birth expectation with the WDEQ-A questionnaire and calculated a mixed ANOVA with the factors group (hypnosis, control) and time point (week 13–28 of pregnancy, week 36–37 of pregnancy). The analysis showed a significant interaction between time point and group *F*(1,124) = 16.07, *p* < 0.001 (see Figure 4). Post-hoc analyses showed that women in the hypnosis group developed a more positive birth expectation at the second time point than women in the control group *t*(124) = 3.71, *p* = 0.002. Furthermore, within the hypnosis group there was a positive change in birth expectation from the first to the second time point *t*(124) = 5.28, *p* < 0.001.

**Figure 5:**
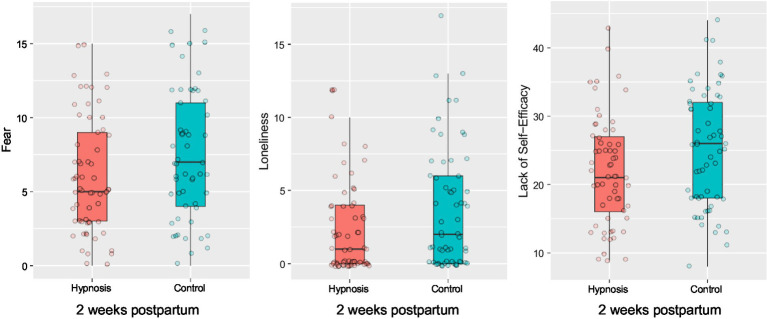
Boxplots for fear, loneliness and self-efficacy subscales depicting significant group differences in birth experience 2 weeks after birth. Women in the hypnosis group showed significantly more positive ratings of fear, loneliness and self-efficacy compared to the control group.

Since the WDEQ-B in German-speaking countries does not represent a unidimensional construct but has subscales, we analyzed birth experience regarding the subscales postulated by König (2019). We found significant effects for the subscales “fear” *t*(113.91) = 2.52, *p* = 0.007, “loneliness” *t*(114.71) = 1.98, *p* = 0.025 and “lack of self-efficacy” *t*(123.63) = 1.68, *p* = 0.047 (see Figure 5). Women in the hypnosis group had a significantly more positive birth experience regarding these three subscales than women in the control group. There was no significant effect on any other subscale (“negative experience”: *t*(120.35) = 0.51, *p* = 0.694; “negative appraisal”: *t*(122.82) = 0.03, *p* = 0.512; “not as it should be”: *t*(116.46) = 0.72, *p* = 0.763). Considering the birth experience as a unidimensional scale did not yield significant group differences *t*(121.73) = 1.13, *p* = 0.13, even when controlling for course use *F*(1,118) = 0.72, *p* = 0.397.

**Figure 6:**
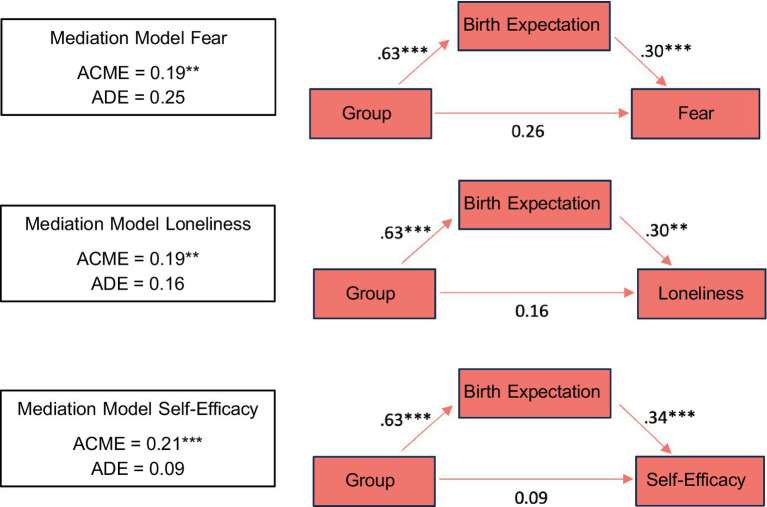
Path diagrams for fear, loneliness and self-efficacy mediation analyses show that birth expectation significantly affected birth experience. Statistics represent standardized regression coefficients. Significance is marked with * (0.01 < *p* < 0.05), with ** (0.001 < *p* < 0.01) and with *** (*p* < 0.001).

To demonstrate the relationship between participation in the intervention, birth expectation and birth experience, and to show that birth expectation has a crucial influence on birth experience, we calculated three separate mediation analyses for fear, loneliness, and lack of self-efficacy, with birth expectation as the mediator (Figure 6). For all analyses, we performed a nonparametric bootstrap with 1,000 simulations to calculate confidence intervals.

The first analysis revealed that the effect of participation in the intervention on fear was mediated through birth expectation ACME = 0.19, 95% CI [0.05, 0.37], *p* = 0.008. The average direct effect, when controlling for the effect of birth expectation on fear, was not significant ADE = 0.25, *p* = 0.176. This pattern represents an indirect-only mediation, making the presence of another mediator unlikely (Zhao et al., 2010). We observed the same pattern for loneliness as the dependent variable of the mediation ACME = 0.19, 95% CI [0.03, 0.39], *p* = 0.004, again with a non-significant average direct effect ADE = 0.16, *p* = 0.352. Similarly, for lack of self- efficacy, we observed an indirect-only mediation ACME = 0.21, 95% CI [0.07, 0.42], *p* < 0.001, with a non-significant average direct effect ADE = 0.09, *p* = 0.602.

### Pain

For pain assessment, we compared both groups on the variables “maximum pain” and “postpartum pain,” each measured on a 10-point scale, and on the variables “pain coping” and “duration of severe pain,” each measured on a 5-point scale.

Although there was a descriptive trend for the variables “pain coping” and “postpartum pain” in favor of the hypnosis group, none of the *t*-tests reached significance [pain coping: *t*(118.73) = 1.43, *p* = 0.078; postpartum pain: *t*(123.71) = 1.44, *p* = 0.081; maximum pain: *t*(102.54) = 0.88, *p* = 0.180; pain duration: *t*(118.6) = 1.16, *p* = 0.876]. Even after statistically controlling for analgetics and medical performed interventions (e.g., caesarean section), no significant group differences were observed [pain coping: *F*(1,103) = 2.84, *p* = 0.094; postpartum pain: *F*(1,103) = 2.28, *p* = 0.134; maximum pain: *F*(1,99) = 1.02, *p* = 0.314; pain duration: *F*(1,103) = 1.46, *p* = 0.230].

### Health and wellbeing

To assess participants’ perceptions of health and wellbeing, we asked about their subjective perception of health and mood over the last 7 days, the baby’s health and the perception of mother–child attachment. At the fourth measurement point, we additionally asked participants how satisfied they were with their life with a newborn, with their sleep, with their baby’s sleep, with wound healing, and to what extent they felt able to cope with the new challenges.

A mixed ANOVA with the factors group (hypnosis, control) and time point (week 13–28 of pregnancy, week 36–37 of pregnancy, 2 weeks after birth, 8 weeks after birth) with Greenhouse–Geisser correction revealed a main effect for the attachment variable, showing that all women developed a stronger sense of attachment over the course of the study, *F*(1,369) = 26.49, *p* < 0.001.

**Figure 7:**
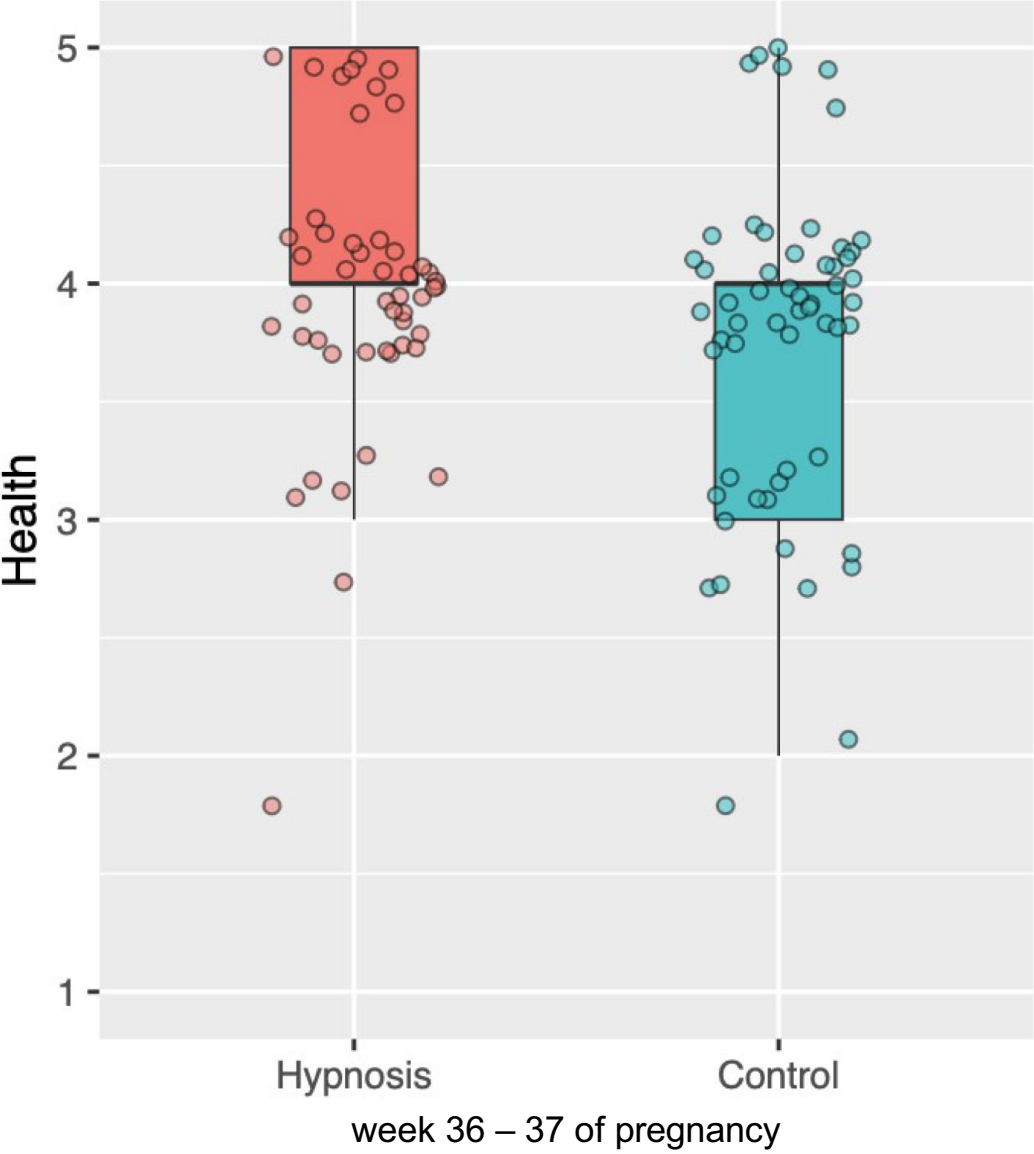
Boxplot depicting the intergroup difference in health assessment at week 36–37 of pregnancy. Women in the hypnosis group rated their health as significantly better compared to the control group.

Furthermore, a separate analysis of each time point showed that the two groups differed at the second time point (Figure 7). Toward the end of their pregnancy, women in the hypnosis group rated their health more positively than women in the control group, *t*(122.17) = 2.962, *p* = 0.002. No further significant effects were observed. Table 2 shows health ratings in both groups for all four measurement points. A significant group difference emerged at the second measurement point, which is shown in Figure 7.

**Table 2:** Health ratings of both groups to each measurement time point.

	Means and standard deviations		Statistics	
Time point	Hypnosis group	Control group	*t*	*p*
Week 13–28	4.02 (sd = 0.67)	3.97 (sd = 0.73)	0.38	0.701
Week 36–37	4.26 (sd = 0.69)	3.89 (sd = 0.73)	2.96	0.004
2 weeks postpartum	3.97 (sd = 0.87)	3.92 (sd = 0.80)	0.34	0.736
8 weeks postpartum	4.02 (sd = 0.82)	3.98 (sd = 0.85)	0.21	0.831

### Additional variables

For exploratory analyses, we examined birth costs, neonatal health scores and data from the ASKU questionnaire and the Mindset and Birth Questionnaire. To assess birth costs, we analyzed the duration of labor, number of medical interventions and administration of analgetics, as only 17 women in the sample provided detailed information on the total cost of childbirth. None of the analyses reached significance [duration of labor: *t*(116.43) = 0.11, *p* = 0.544, number of interventions: *t*(123.99) = 0.19, *p* = 0.424, analgetics: *t*(122.87) = 0.19, *p* = 0.577].

Similarly, analyses of the APGAR score, 5 and 10 min after birth and umbilical artery pH as neonatal health characteristics showed no significant group difference [APGAR 5 min after birth: *t*(112) = 0.90, *p* = 0.370; APGAR 10 min after birth: *t*(111.85) = 0.75, *p* = 0.455; umbilical artery pH: *t*(101.12) = 1.15, *p* = 0.251].

The same applies to the examination of self-efficacy expectations using the ASKU questionnaire *t*(83.32) = 0.43, *p* = 0.670, and the analysis of the birth-related mindset using the Mindset and Birth Questionnaire *t*(95.25) = 0.83, *p* = 0.410.

### Additional analyses within the hypnosis group

**Figure 8:**
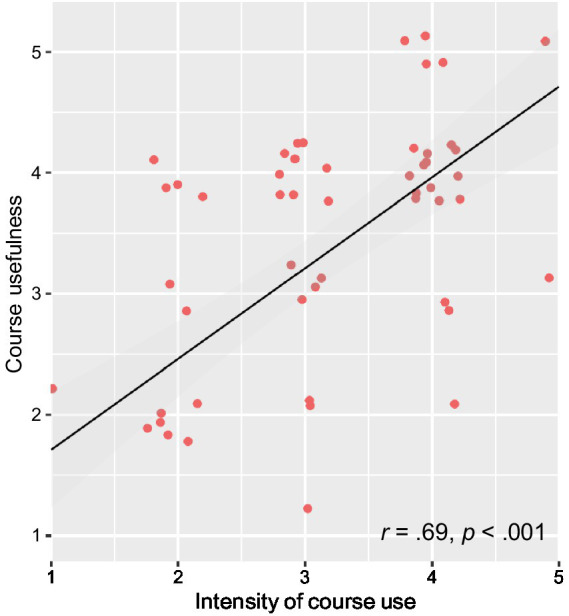
Relationship between the intensity of course use and the perception of the course as overall useful measured 8 weeks after birth. The more intensively the course was used, the greater the perceived benefit.

To get an impression of which women within the hypnosis group particularly benefited from the intervention, we additionally calculated Pearson product–moment correlations between subjective benefits of the course and the intensity of course use. Subjective benefits included, for example, the evaluation of the overall utility of the course, its usefulness for the time before, during, and after childbirth, as well as perceived advantages for anxiety and pain reduction. Correlations were moderate to high ranging from *r* = 0.32, *p* = 0.02 to *r* = 0.69, *p* < 0.001. Women who used the course more intensively perceived the benefits of the course to be greater. Figure 8 shows the correlation between course use intensity and the perception of the course as overall useful 8 weeks after birth (*r* = 0.69).

## Discussion

The aim of the present study was to evaluate the hypnosis online course “The Peaceful Birth.” We wanted to know if the course alters women’s expectations about childbirth, their fear surrounding it, stress levels, physical well-being, and the actual birth experience itself. Therefore, we surveyed a hypnosis group (*n* = 65) and a control group (*n* = 61) at four time points. Our data show that the hypnosis online course significantly improved birth expectation, significantly reduced stress and fear, and significantly improved health-related well-being in anticipation of childbirth compared to the control group. Furthermore, women who used the hypnosis online course perceived the birth experience itself as significantly more positive regarding fear, loneliness and self-efficacy compared to the control group.

Future studies can extend these findings and include an active control condition, exclude potential bias, add data from treating institutions in addition to self-reports, add a measure of hypnotizability and conduct an intention-to-treat analysis.

With these results, our study adds to the body of literature showing that hypnosis is an effective method to improve the outlook toward birth (Streibert et al., 2015; Werner et al., 2013) and the birth experience itself (Atis and Rathfisch, 2018). It is one of the first studies in the field of clinical hypnosis to evaluate the causal link between birth expectation and birth experience. It thus makes an important contribution to hypnosis and maternal health research.

We assume that reasons for the improved anticipation of birth can be found in the transactional stress model (Lazarus and Folkman, 1984). According to this model, stress arises when a situation is perceived as threatening and the individual perceives its abilities and resources as insufficient to cope with the situation. As childbirth is often associated with fear- inducing cognitions such as the possible risk of complications, loss of control or severe pain, the result is an increased experience of stress. Fear and stress can be reduced either by reducing negative cognitions about birth and correspondingly perceiving the situation as less threatening, or by promoting confidence in one’s own coping resources. Similarly, Antonovsky’s concept of salutogenesis emphasizes the promotion of health by strengthening coping resources and fostering a sense of coherence, enabling individuals to perceive challenging situations like childbirth as comprehensible, manageable, and meaningful (Antonvsky et al., 1997; Prinds et al., 2022).

Our intervention promotes positive suggestions to strengthen women’s confidence in their internal resources (e.g., their own abilities) and in external resources (e.g., the support of the medical staff). In addition, the hypnotherapeutic method of time progression trains the imagination of an optimal birth and necessary steps to reach this goal. In this way, women learn to experience themselves as competent and capable. In another study investigating the efficacy of hypnosis for stress coping, a qualitative analysis of a focus group found that participants perceived hypnosis as a tool that “increased their feeling of control in demanding situations, especially due to the activation of resources to better cope with stressful situations” (Fisch et al., 2020).

On the other hand, the hypnosis online course also reduced anxiety-related cognitions by the knowledge transfer in the video modules. Previous research has demonstrated that imparting knowledge, in addition to hypnosis, has a fear-reducing effect (Hosseini et al., 2018). This may lead to a less threatening perception of childbirth.

In our study, we show that the hypnosis intervention significantly improved women’s health assessment at the end of pregnancy. That is in line with other studies suggesting that the use of hypnosis-based interventions has a positive effect on mental health (Dobbin et al., 2004), as well as on physical health (Barling and Raine, 2005). It is possible that the reduction of negative cognitions and the increase in coping skills is the reason why women who participated in the intervention felt significantly better and had a significantly more positive assessment of their health. However, more research is needed to investigate this relationship.

Regarding the birth experience itself, we found that during birth, mothers in the hypnosis group experienced lower levels of fear, loneliness, and increased self-efficacy. This effect was not only found in the quantitative analysis, but some women also used the open comments field to state that they had a strong sense of self-efficacy during birth and that the course had helped them to make self-determined decisions during birth. Our mediation analyses show that the reduction of stress and fear as well as the promotion of positive expectations prior to birth had a significant impact on birth experience. Accordingly, positive anticipation resulted in a more positive perception of birth. This finding is also in line with previous research which shows that more positive birth expectations lead to greater satisfaction with birth experience (Green et al., 1990, 1998). To our knowledge, our study is the first to demonstrate this causal relationship in the context of a hypnosis-based intervention. Therefore, we were able to show that using hypnosis for altering mental constructs like cognitions and expectations has an impact on the perception of unique and challenging situations like birth.

We did not discover a significant influence of the hypnosis intervention on the sensation of pain during birth. As similar studies have suggested such an effect, it is necessary to discuss possible reasons for this finding. One potential explanation is that the intervention did not affect pain intensity, but valence. This explanation is based on a study by Abbasi et al. (2009) which showed that pain sensation can be reinterpreted as pressure sensation due to a hypnosis intervention. It is possible that women in both groups experienced a similar intensity of pain, but women in the hypnosis group perceived this intense pain as less negative. For example, one woman of the hypnosis group described her experience as follows: “Even though I still had a very painful birth, I did not experience it negatively.” Another explanation can be found in the methodological limitations of the study. Due to a two-week delay between the birth and the enquiry about the birth experience, memories of the pain sensation may have been distorted.

Dropout rates were similar in both groups and about 25% from first to last measurement. Other reasons for exclusion were for example that 14 women in the control group used “The Peaceful Birth” privately.

### Limitations

The study design resulted in a few methodological limitations that need to be discussed.

The control group knew that they are taking part in a study that evaluates the efficacy of a birth preparation program and that they are in the control group. That means, the control group was unblinded regarding group assignment.

The control group did not receive a placebo intervention, so they might have perceived the intensive work with “The Peaceful Birth” as a more intensive contact with the experimenters. Please note that all participants received the same four questionnaires during their pregnancy and afterwards, so they were treated equally concerning the level of contact with experimenters.

The results are based on self-reports only, we did not contact the treating institutions. It is possible that we would have obtained more detailed data from the treating institutions.

We did not measure hypnotizability in our participants, so we cannot relate the level of hypnotizability to the effect of the hypnosis intervention.

We did not ask about adverse events that might have occurred during the use of “The Peaceful Birth.”

We used the calculated date of birth to send out the questionnaires at all measurement points. However, for many women, the actual date of birth differs from the calculated date. In Germany, around 60% of babies are born within a week before or after the calculated date, but very few are born exactly on the calculated date (Weiss et al., 2014). This means that the recordings of the third and fourth time point, 2 and 8 weeks after birth, vary by a few days or weeks between individuals. We assume that the time directly after the birth is a very intense time for women, so that delays in answering the questionnaires may also have affected the way the questionnaires were answered. Even if we assume that these variations occurred in both groups and are therefore unlikely to affect group differences, this limitation should still be taken into account.

In addition, as mentioned above, the third time point is intended to record the birth experience, but was not recorded immediately after birth, but with a delay of 2 weeks. Due to various physiological, hormonal and psychological processes that significantly influence the time after birth (Lasch, 2017), memories of the birth experience may have been distorted (Dahan, 2023). This methodological limitation is due to ethical reasons and was therefore unavoidable. It is possible that the recall of several memories, for instance, pain memories may have been affected by this limitation.

In our sample, the majority of women gave birth in hospitals. Therefore, we did not differentiate between hospital and home birth. The literature suggests that a planned home birth is associated with a more positive perception of birth (Janssen et al., 2009). In our control group, 5 women gave birth at home, while in our hypnosis group, only 2 women gave birth at home. Therefore, we assume that possible positive effects of giving birth at home would have favored the control group in our sample and the improvements in the hypnosis group are due to our hypnosis intervention.

A final important point is the limited control we had over how the women used the hypnosis online course. As we made the course available to the women for independent use, we could not ensure that each woman listened to the same audio hypnosis. We assume that there were large inter-individual differences here. We found that the more intensely women used the hypnosis online course, the higher they rated their subjective benefit. Accordingly, it is plausible to assume that some effects might have been higher if the use of the hypnosis intervention had taken place in a more controlled setting. However, the design that we chose is much more likely to reflect reality, and therefore displays a high ecological validity.

## Conclusion and implications

Our study shows that using a hypnosis-based intervention such as “The Peaceful Birth” helps women to improve birth expectations and birth experiences. In particular, it is helpful to reduce anxiety, relieve stress and strengthen confidence in one’s own coping skills. We show that these are crucial factors for enhancing the experience of childbirth. It furthermore shows that the method of hypnosis can be learned through low-threshold programs that do not require the presence of a hypnotist. In this way, mothers learn to see themselves as capable of coping with challenging situations like childbirth.

For future research, we suggest further exploration of hypnosis-based birth preparation. As there are already many commercial programs on the market, their efficacy should be investigated. This could lead to greater financial support from health insurance companies, thus enabling more women to have a more positive birth experience. It would also make it possible to distinguish between courses that are scientifically founded and those that are not. Further research into the effects we found in our study would also be desirable. For example, we suggest a more concrete evaluation of the improved health parameters before childbirth, but also beyond. Additionally, the efficacy of our and other hypnosis interventions regarding the experience of pain should be investigated further.

In conclusion, we claim that hypnosis is a powerful tool for improving the birth experience of expectant mothers and minimizing the number of women who experience birth as negative or even traumatic. We conclude that hypnosis courses like “The Peaceful Birth” make a substantial contribution to maternal health care.

## Data Availability

The datasets presented in this study can be found in online repositories. The names of the repository/repositories and accession number(s) can be found at: https://zenodo.org/records/10160390.

## Footnotes

^1^ https://zenodo.org/records/10160390
